# Osteoconductive properties of upside-down bilayer collagen membranes in rat calvarial defects

**DOI:** 10.1186/s40729-021-00333-y

**Published:** 2021-06-07

**Authors:** Balazs Feher, Karol Ali Apaza Alccayhuaman, Franz Josef Strauss, Jung-Seok Lee, Stefan Tangl, Ulrike Kuchler, Reinhard Gruber

**Affiliations:** 1 Department of Oral Biology, University Clinic of Dentistry, Medical University of Vienna, Sensengasse 2a, 1090 Vienna, Austria; 2 Clinic of Reconstructive Dentistry, Center of Dental Medicine, University of Zurich, Zurich, Switzerland; 3 Department of Conservative Dentistry, Faculty of Dentistry, University of Chile, Santiago, Chile; 4 Department of Periodontology, Research Institute for Periodontal Regeneration, College of Dentistry, Yonsei University, Seoul, Korea; 5 Core Facility Hard Tissue and Biomaterial Research, Karl Donath Laboratory, University Clinic of Dentistry, Medical University of Vienna, Vienna, Austria; 6 Austrian Cluster for Tissue Regeneration, Vienna, Austria; 7 Department of Oral Surgery, University Clinic of Dentistry, Medical University of Vienna, Vienna, Austria; 8 Department of Periodontology, School of Dental Medicine, University of Bern, Bern, Switzerland

**Keywords:** Collagen membranes, GBR, GTR, Micro-computed tomography, Histology, Histomorphometry, Preclinical research

## Abstract

**Background:**

Bilayer collagen membranes are routinely used in guided bone/tissue regeneration to serve as osteoconductive scaffolds and prevent the invasion of soft tissues. It is recommended to place the membranes with their dense layer towards the soft tissue and their porous layer towards the bony defect area. However, evidence supporting this recommendation is lacking. This study aimed to determine whether the alignment of bilayer collagen membranes has an effect on bone regeneration.

**Methods:**

In two groups of ten male Sprague-Dawley rats each, a 5-mm calvarial defect was created. Thereafter, the defect was randomly covered with a bilayer, resorbable, pure type I and III collagen membrane placed either regularly or upside-down (i.e., dense layer towards bone defect). After 4 weeks of healing, micro-computed tomography (μCT), histology, and histomorphometry of the inner cylindrical region of interest (4.5 mm in diameter) were performed to assess new bone formation and the consolidation of the collagen membrane in the defect area.

**Results:**

Quantitative μCT showed similar bone volume (median 8.0 mm^3^, interquartile range 7.0–10.0 vs. 6.2 mm^3^, 4.3–9.4, *p* = 0.06) and trabecular thickness (0.21 mm, 0.19–0.23 vs. 0.18 mm, 0.17–0.20, *p* = 0.03) between upside-down and regular placement, both leading to an almost complete bony coverage. Histomorphometry showed comparable new bone areas between the upside-down and regularly placed membranes, 3.9 mm^2^ (2.7–5.4) vs. 3.8 mm^2^ (2.2–4.0, *p* = 0.31), respectively. Both treatment groups revealed the same regeneration patterns and spatial distribution of bone with and without collagen fibers, as well as residual collagen fibers.

**Conclusions:**

Our data support the osteoconductive properties of collagen membranes and suggest that bone regeneration is facilitated regardless of membrane layer alignment.

**Supplementary Information:**

The online version contains supplementary material available at 10.1186/s40729-021-00333-y.

## Background

Regenerative treatment strategies routinely involve the use of collagen membranes to prevent the invasion of non-osteogenic soft tissues [1–4]. Collagen membranes are supposed to consist mainly of collagen types I and III [5] and are typically resorbable [6]. Some porcine peritoneum-derived collagen membranes feature a bilayer design with a dense layer facing the soft tissue and a porous layer covering the defect area. The putative function of the dense layer is to keep the soft tissue at a distance while the porous layer is infiltrated by osteogenic cells originating from the bony defect site [7–9]. In addition to their passive function as occlusive barriers with a porous part supporting osteogenic cell migration [10, 11], collagen membranes are supposed to directly affect the cellular aspects of regeneration [12, 13], including the adsorption of locally produced growth factors [12–14]. Considering that collagen membranes are heterogenous and bone regeneration is initiated at the defect margins, it is recommended to place the dense layer towards the soft tissue and the porous layer towards the bony defect area. Evidence supporting this clinical recommendation, however, is lacking.

We have recently shown that bone forms inside peritoneum-derived collagen membranes that underwent lyophilization [15, 16]. Collagen membranes can thus serve as mineralization substrate [17], acting as osteoconductive scaffolds. Calvarial defect sites are rich in osteoblast progenitor cells [18] that could be derived from the periosteum [19], the capillaries [20], or the dura mater [21] and contribute to new bone formation. Based on the supposedly different functions of the dense layer and the porous layer of collagen membranes, we raise the question whether bone regeneration is affected by the alignment of the layers. If the membrane were to be placed “upside-down” (i.e., dense layer facing the defect), the porous layer would be isolated from the defect but could potentially be repopulated by cells from the periosteum instead. It is thus reasonable to hypothesize that the upside-down collagen membrane is also capable of supporting the migration of osteogenic cells originating from the elevated periosteum in rat calvarial defects.

Support for this hypothesis comes from preclinical [22, 23] as well as clinical studies [24, 25] showing better outcomes after using perforated collagen membranes for guided bone/tissue regeneration. These better outcomes suggest a beneficial role of cellular migration from the periosteum into the defect site. Here, we raised the question whether it is possible to facilitate in upside-down membranes a similar regeneration as in regularly placed membranes. To this end, we used unperforated membranes so as not to deliberately connect the periosteum with the defect site. Based on a combination of micro-computed tomographic (μCT) imaging and histological and quantitative histomorphometric analyses of undecalcified thin-ground sections, we analyzed the bone formation within collagen membranes that had been placed either regularly or upside-down on a critical size calvarial defect in the rat.

## Methods

### Experimental animals

Experimental protocols followed ARRIVE guidelines and were approved by the Medical University of Vienna ethical review board for animal research as well as the Austrian Federal Ministry of Education, Science, and Research (No. BMWFW-66.009/0217-WF/V/3b/2017). Twenty 10-week-old male Sprague-Dawley rats (300–400 g) were used in this study. Rats were housed in groups of three in cages with various enrichment materials, including nesting and gnawing materials, as well as plastic shelters. Rats were maintained on a 12-h day/night cycle and received water as well as a regular diet ad libitum. Preoperatively, a computer algorithm based on atmospheric noise randomized rats into two treatment groups: (i) calvarial defect coverage using a bilayer collagen membrane with its dense layer facing towards the defect (regular group) or (ii) away from the defect (upside-down group). Surgeons remained blinded to treatment allocation until the membrane needed to be placed on the defect, examiners remained blinded until after analysis and all other personnel working with the animals remained blinded during the entire study.

### Surgery

Rats were anesthetized by ketamine 100 mg kg^−1^ i.m. and xylazine hydrochloride 5 mg kg^−1^ i.m., and a 5-mm standardized critical size calvarial defect was created, as previously described [15]. In short, a circular bone disk was removed from the left parietal bone using a trephine drill with an outer diameter of 5 mm. The created ≈ 20 mm^2^ critical size defect was covered with a commercially available, bilayer pure collagen type I and III membrane placed either regularly or upside-down, in accordance with the randomized treatment allocations. The membrane overlapped the defect perimeter by at least 1 mm at every point. The membrane was not fixed to the bone. Wounds were closed in two layers with resorbable USP 5–0 sutures. Butorphanol 1.25 mg kg^−1^ s.c. and meloxicam 0.15 mg kg^−1^ s.c. were used to control postoperative pain. Rats were sacrificed after 4 weeks of healing by an intracardial overdose of thiopental.

### Micro-computed tomography

Heads were fixed in phosphate-buffered formalin. Ex vivo μCT scans were performed at 90 kV and 200 μA with an isotropic resolution of 17.2 μm and an integration time of 500 ms. The images were standardized so that the drill direction was oriented along the *z*-axis with the defect in the approximate center of the image. The region of interest (ROI) was defined as the right circular cylinder aligned to the defect center with a base of *r* = 2.25 mm parallel to the defect area and an individually set *h* for each scan to get smallest possible volume that still contains all new bone (2.8 mm ≤ *h* ≤ 3.1 mm). ROI were automatically positioned and segmented from the μCT images with an ImageJ ruleset developed by us and defect coverage, new bone volume, and trabecular thickness were measured [26].

### Histology and histomorphometry

**Fig. 1:**
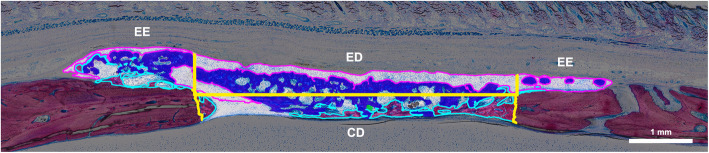
Regions of interest and tissue classes in the histomorphometric analysis. The defect area is divided (yellow) into three regions of interest: a central defect area (CD) between the defect edges, an ectocranial defect area (ED) directly above the central defect area, and two ectocranial defect edges (EE) laterally to the ectocranial defect area in both directions. Bone with collagen (royal blue fill), bone without collagen (light blue), and mineralized collagen fibers (pink) are quantified

Samples were dehydrated with ascending alcohol grades and embedded in light-curing resin. Blocks were further processed using cutting and grinding equipment. Thin-ground sections from all samples were prepared in a plane parallel to the sagittal suture and through the center of the defect. The thin-ground sections were then stained with Levai-Laczko dye. The stained slices were scanned using a digital virtual microscopy system with a × 20 objective resulting in a resolution of 0.32 μm px^−1^ and then evaluated. Three ROI were defined: the central defect area (CD) containing the space of the removed parietal bone, the ectocranial defect area (ED) containing the fixed-width space between the CD and the periosteum, and the ectocranial defect edges (EE) containing the ectocranial space adjacent to the ED on both sides. In all ROI, respective areas of bone with and without collagen fibers, soft tissue, mineralized fibers, residual collagen fibers, and brain prolapse were measured (Fig. 1).

### Statistics

Data are presented as medians and interquartile ranges unless stated otherwise. A sample size of 10 animals per group was calculated based on our recent work [16] to achieve 1 − *β* = 0.80 and *ɑ* = 0.05, assuming unequal variances and Glass’s *Δ* = 1.25. Statistical analysis was based on quantitative μCT and histomorphometry. For μCT, primary outcomes were new bone volume [mm^3^] and trabecular thickness [mm] and the secondary outcome was defect coverage [%]. For histomorphometry, primary outcomes were bone area with or without fibers [mm^2^] and secondary outcomes were areas of soft tissue, mineralized fibers, residual collagen fibers, and brain prolapse [mm^2^]. Outcomes were compared with Mann-Whitney *U* test due to the small sample size even though some variables passed the Shapiro-Wilk test for normality.

## Results

### Micro-computed tomography

**Fig. 2:**
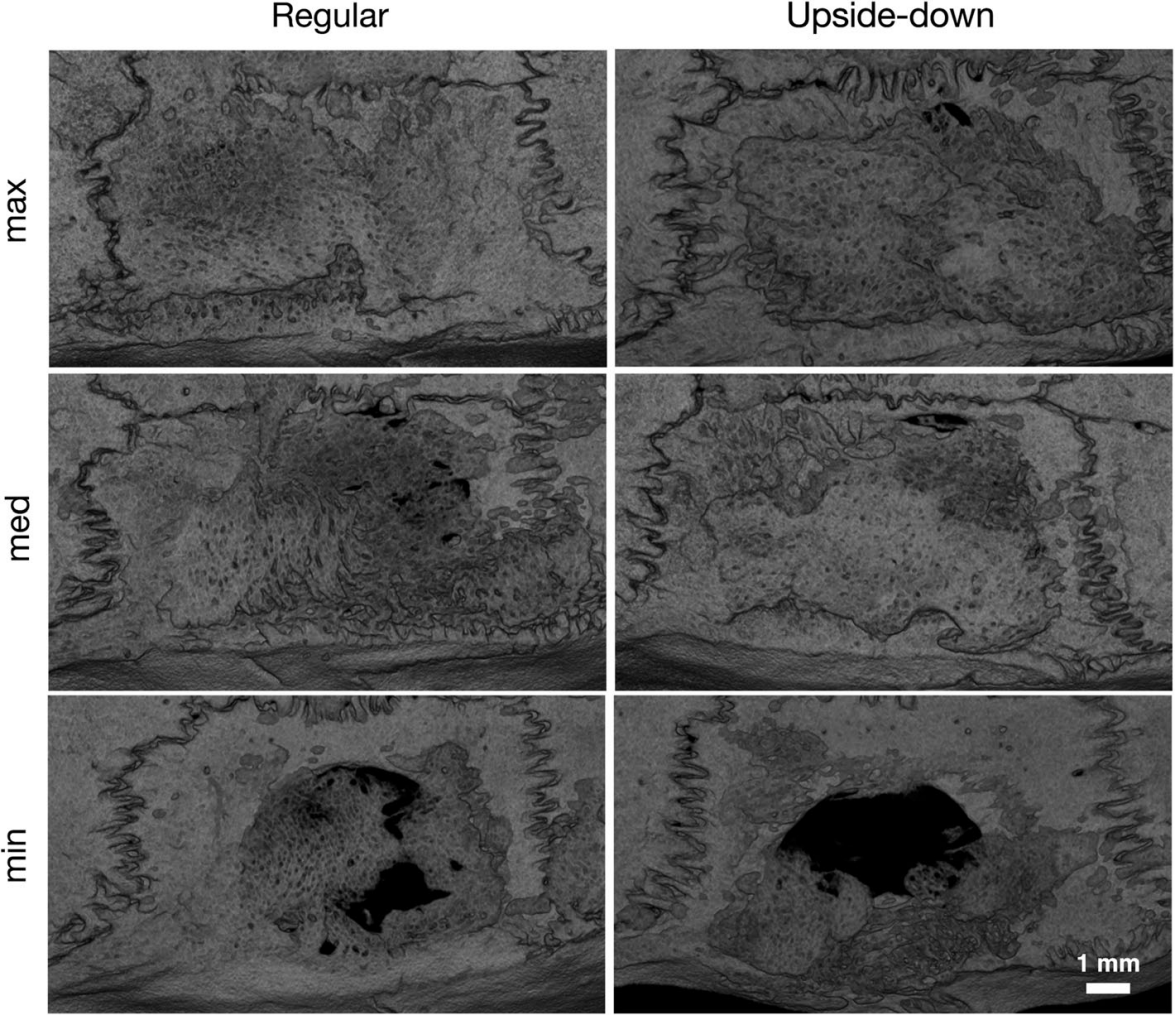
Ex vivo μCT overview of the calvarial defect anatomy after 4 weeks of healing. Critical size defects (*d* = 5 mm) were covered using native resorbable bilayer collagen membranes placed regularly or upside-down. Representative samples of minimal, median, and maximal bone coverage of the defect in the respective groups are shown. Anterior is left. μCT, micro-computed tomography

**Fig. 3:**
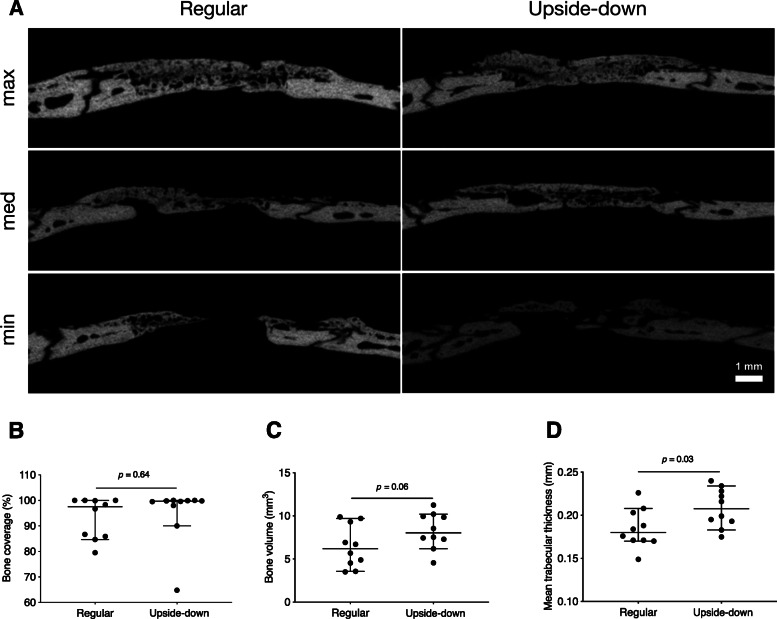
Ex vivo μCT results. **a** Lateral view of defects covered with regularly placed or upside-down membranes. Representative samples of minimal, median, and maximal bone coverage of the defect in the respective groups are shown. **b** Relative bone coverage of the defect (*p = 0.*64). **c** Total new bone volume inside the ROI (*p = 0.*06). **d** Mean trabecular thickness (*p = 0.*03, bars and whiskers represent medians and interquartile ranges, all *p*-values using Mann-Whitney *U* test). μCT, micro-computed tomography

We previously showed that lyophilized collagen membranes possess osteoconductive properties in standardized calvarial defect models [15, 16]. However, the osteoconductive properties of native collagen membranes remained to be tested under these conditions. We covered 5-mm critical size defects in the left parietal bone with either regularly placed or upside-down membranes. Considering the bilayer membrane structure with an occlusive and a spongy layer, we hypothesized that the osteoconductive properties are affected by the membrane alignment. To this aim, we first assessed the bone coverage of the calvarial defect using μCT. Notably, both regular and upside-down membranes led to a virtually complete bone coverage of the defects (Figs. 2 and 3a).

Quantitative analysis showed that the ≈ 16 mm^2^ circular defect area inside the ROI was covered with new bone, amounting to 99.7% (96.0–100.0) in the upside-down group and 97.5% (85.6–100.0) in the regular group (*p* = 0.64) (Fig. 3b). We next calculated whether the overall volume of new bone was affected by the alignment of the collagen membranes. We found a tendency towards higher new bone volume in the upside-down group compared with the regular group, 8.0 mm^3^ (7.0–10.0) vs. 6.2 mm^3^ (4.3–9.4, *p* = 0.06). Consistently, mean trabecular thickness was significantly higher in the upside-down group compared with the regular group, 0.21 mm (0.19–0.23) vs. 0.18 mm (0.17–0.20, *p* = 0.03) (Fig. 3c, d). Despite this significant difference, these results imply that membranes placed upside-down can lead to similar bone regeneration compared with regularly placed membranes.

### Histology

**Fig. 4:**
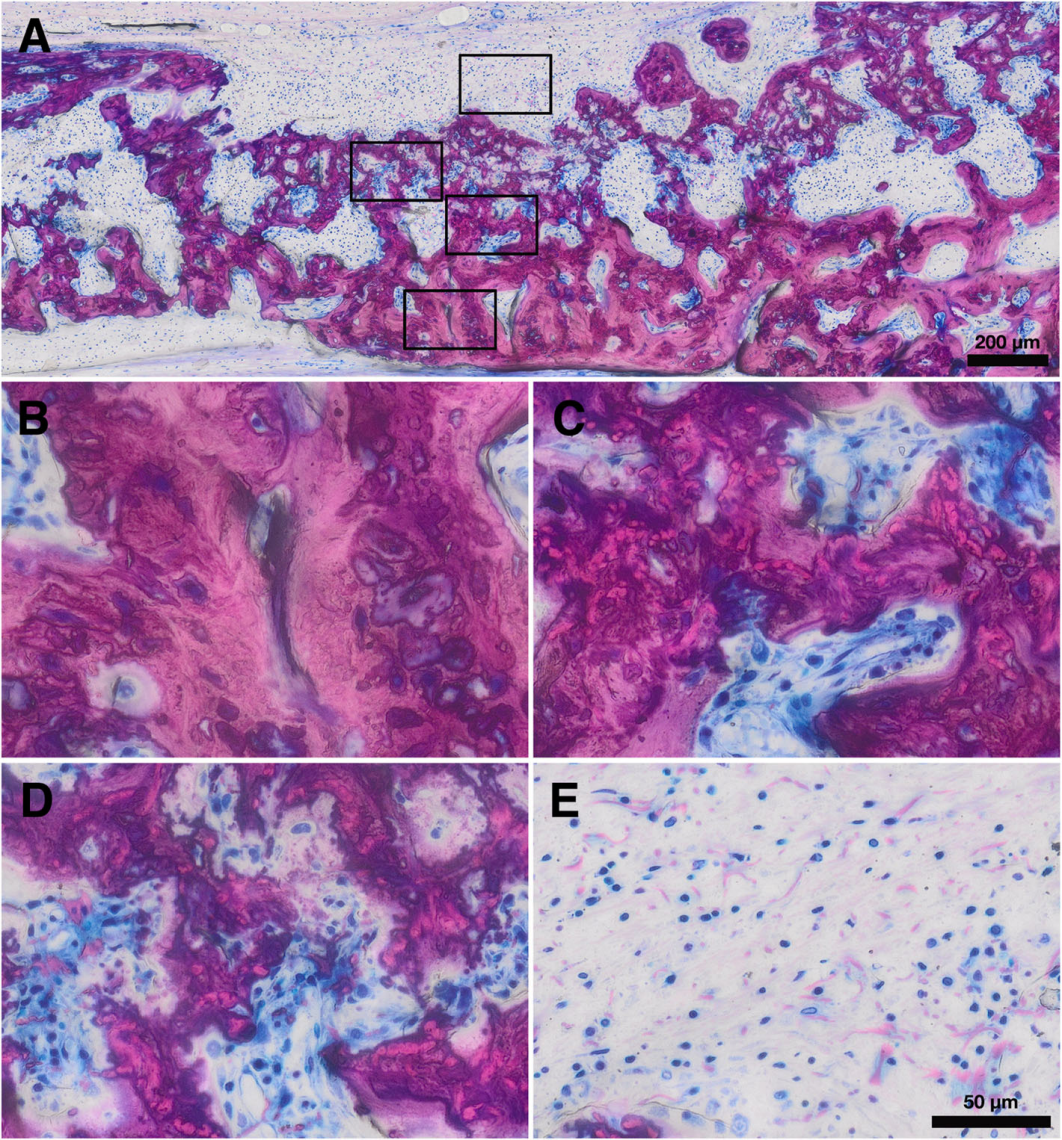
Histological overview of a defect treated with a regular membrane. **a, b** Bone with collagen. **c, d** Bone without collagen. **e, f** Mineralized collagen fibers

**Fig. 5:**
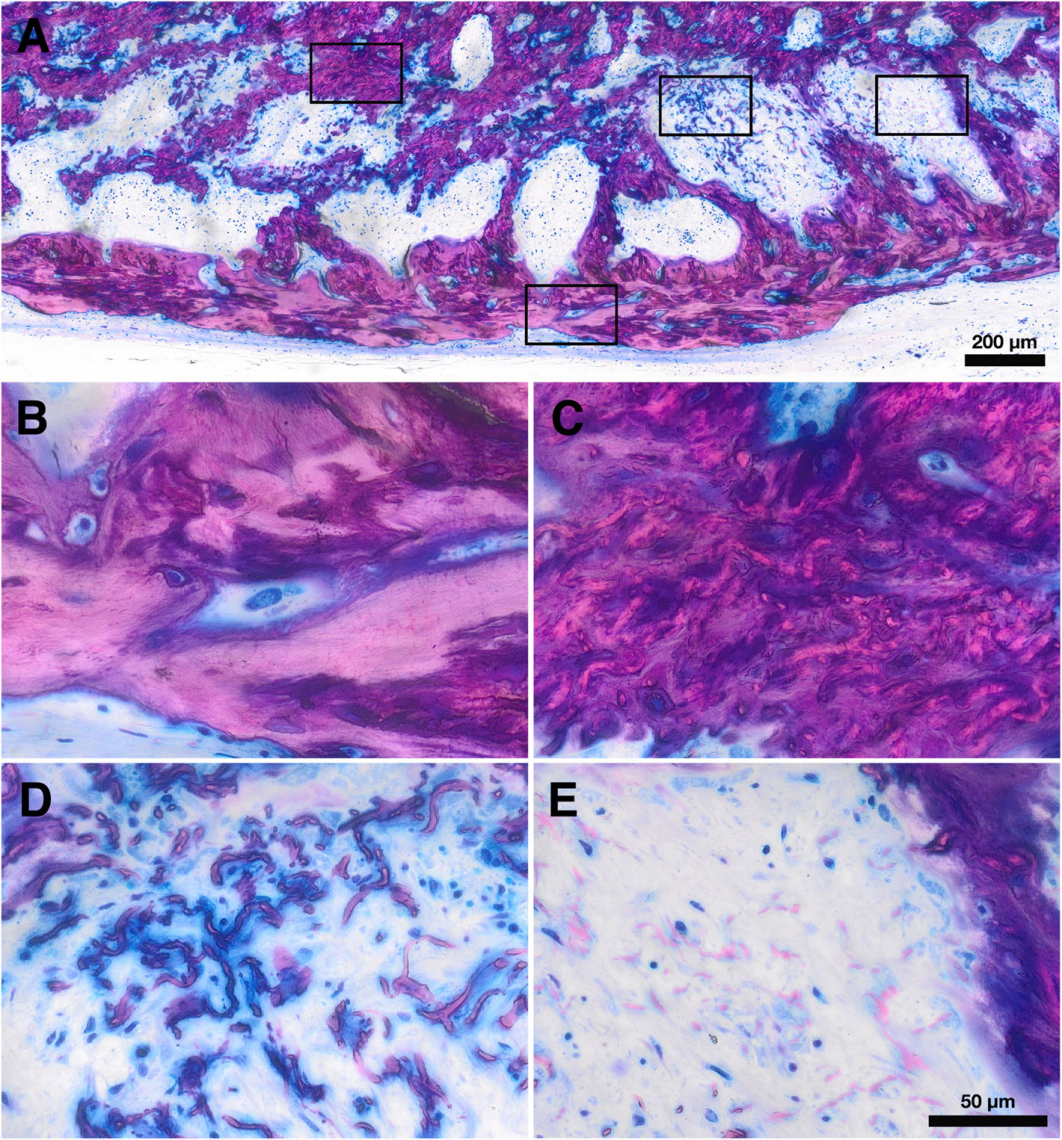
Histological overview of a defect treated with an upside-down membrane. **a, b** Bone with collagen. **c, d** Bone without collagen. **e, f** Mineralized collagen fibers

We next obtained thin-ground sections stained with Levai-Laczko dye to perform a descriptive histological analysis. Our approach allowed us to examine the three main tissue types of interest (bone with collagen, bone without collagen, and residual collagen fibrils) in the regular and upside-down groups (Figs. 4 and 5). We could not observe discernible differences between the two groups regarding the tissue areas and distribution patterns. We found a large portion of the new bone in the ectocranial area and a smaller portion in the central defect area. In both groups, a majority of new bone showed embedded collagen fibers. New bone with embedded collagen new bone was primarily found in the ectocranial area. New bone without collagen fibers was generally found in the central defect area, in close proximity to the defect edges or the dura mater. Between the periosteum and the new bone with collagen fibers, a discrete layer of collagen fibers was visible without new bone formation. Overall, descriptive histology revealed that both the evaluated areas and the spatial relationships of the different tissues were comparable between the regular and upside-down groups.

### Histomorphometry, total bone

**Fig. 6:**
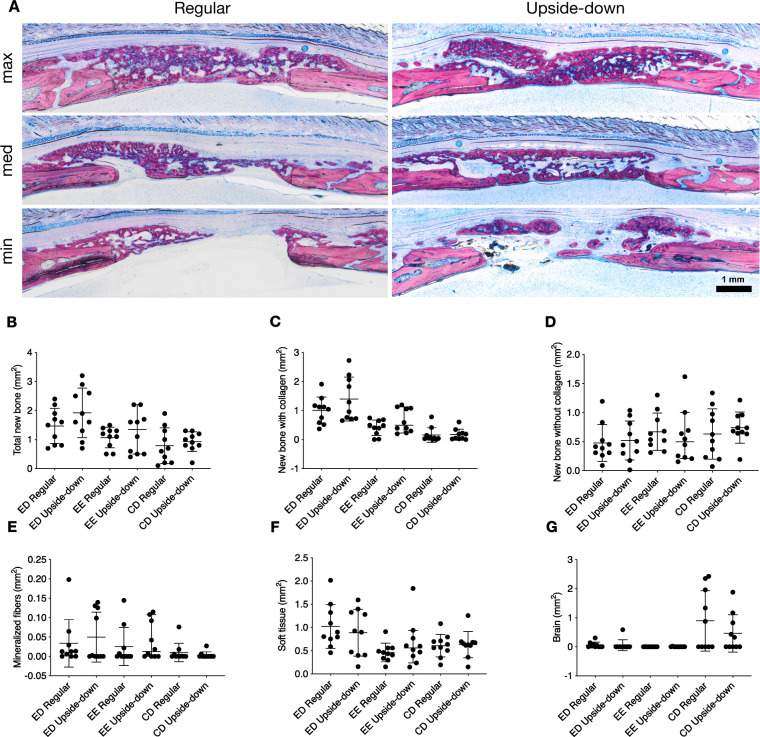
Histological and histomorphometric results. **a** Histological overview of the defect anatomy after 4 weeks of healing. Quantitative histomorphometric analysis of **b** total new bone, **c** new bone with collagen, **d** new bone without collagen, **e** mineralized fibers, **f** soft tissue, and **g** brain prolapse in all groups and regions of interest (bars and whiskers represent medians and interquartile ranges). CD, central defect area; ED, ectocranial defect area; EE, ectocranial defect edges

To further investigate these findings and quantify the various tissue areas within and around the defect, we performed a histomorphometric analysis. We differentiated three ROI (CD, ED, and EE) and six tissue types (bone with collagen fibers, bone without collagen fibers, soft tissue, mineralized fibers, residual collagen fibers, and brain prolapse) (Fig. 1). An overview of representative histological samples from the different groups is shown in Fig. 6a. Only the total new bone area tended to differ slightly between upside-down and regular groups, 3.9 mm^2^ (2.7–5.4) vs. 3.8 mm^2^ (2.2–4.0, *p* = 0.31) (Fig. 6b). Most of the new bone area, 75% in the upside-down and 80% in the regular group, was found in the ectocranial (ED and EE) ROI. In these ROI, we could also observe largely similar new bone areas in the upside-down and regular groups, 3.4 mm^2^ (1.7–4.6) vs. 2.6 mm^2^ (2.0–3.2, *p* = 0.40) (Fig. 6b). While total, ED, and EE new bone area all tended to be higher in the upside-down compared with the regular group, the differences were not significant. These results suggest that membranes placed upside-down lead to similar degrees of bone regeneration compared with regular membrane alignment.

### Histomorphometry, bone with and without collagen

Next, we took advantage of the visualization of the remaining collagen fibrils now entombed in the new bone to examine whether the changes in bone formation are linked to the presence of collagen fibrils. We first measured overall new bone area with collagen fibers in all ROI. New bone area with collagen was comparable between upside-down and regular groups, 1.7 mm^2^ (1.2–3.5) vs. 1.7 mm^2^ (0.9–2.1, *p* = 0*.*39), respectively (Fig. 6c). Focusing on ED and EE, we could again observe comparable degrees of bone formation between upside-down and regular groups, 1.5 mm^2^ (1.1–3.3) vs. 1.5 mm^2^ (0.9–1.9, *p* = 0.44) (Fig. 6c). We observed virtually no bone with collagen fibers in CD. Moreover, there were no discernible differences related to collagen membrane alignment in bone without visible collagen fibers (Fig. 6d)**.** Taken together, these findings suggest that treating calvarial defects with membranes placed upside-down leads to a highly comparable distribution of new bone tissue compared with regular membrane alignment.

### Histomorphometry, mineralized collagen fibers

Previously, we identified regions with collagen fibers staining positive for mineralization but no visible bone-forming osteoblasts [15]. Here, we found similar areas of mineralized collagen fibers, mostly located in the ectocranial regions, between the new bone with collagen fibers and the periosteum. Upside-down and regular groups showed no differences with regard to the areas occupied by mineralized collagen fibers (Fig. 6e). At least in a rat calvarial model, collagen fibers appear to undergo mineralization. These findings suggest that the processes of collagen mineralization occur regardless of membrane alignment.

## Discussion

Collagen membranes placed upside-down allowed a similar degree of bone regeneration in calvarial defects compared with regular membrane placement. Our data show similar new bone volume and trabecular thickness in defects treated with upside-down membranes; radiological defect coverage as well as histomorphometric parameters were largely similar between the two groups. These findings suggest that the porous membrane layer need not be oriented towards the defect to facilitate new bone formation.

The present study is the first to compare bone ingrowth into native regular with upside-down collagen membranes. Nevertheless, our findings relate to those of others testing the impact of modified (e.g., perforation alone or in combination with growth factors) collagen membranes on tissue regeneration [24, 25, 27–30]. In periodontal intrabony defects, perforated membranes led to improved clinical parameters compared with regular membranes [30]. In one preclinical study, a perforated membrane was used upside-down to enable the loading of the porous layer with bone morphogenetic proteins to be delivered to the periosteum; native regular or upside-down membranes were not tested [31]. Here, we used an intact native membrane and placed it upside-down on the defect so that its dense layer faces the defect itself and its porous layer faces the periosteum. While our method ostensibly lets cells from the periosteum migrate into the porous layer, the defect itself remains completely covered by the dense layer. However, we found comparable degrees of bone regeneration without membrane perforation, suggesting that native bilayer collagen membranes can facilitate bone regeneration regardless of layer alignment.

The putative function of the porous layer is to provide a matrix for migrating osteogenic cells. We observed a trend towards new bone volume and trabecular thickness in defects where the porous layer faced the periosteum. However, we can neither locate the new bone to either the porous or the dense membrane layer nor do we conclude that the periosteum serves as the source of the osteogenic cells in either of the membrane positions. Based on the rather dense arrangement of the collagen fibers entombed in the bone and scanning electron microscopic observations of native membranes [9], we can speculate that bone formation mainly occurs in the dense part of the collagen membrane. It is also plausible that the porous layer is rapidly degraded by collagenases, leaving only the dense layer as an osteoconductive scaffold. Obviously, it is relevant to understand the osteoconductive properties of the dense and the porous layers as they provide information on the future development of osteoconductive scaffolds on a collagen basis. Certainly, our speculations also raise further questions in terms of the effects of the various membrane layers. Theoretically, it would be possible to eliminate the dense layer completely or even use a modified membrane with two porous layers. One possible advantage would be the rapid migration of osteogenic cells. This would have to be weighed against the possible disadvantage of fast soft tissue invasion. However, we cannot discuss the possible role of the dense layer in modulating osteoconductivity as long as the origin of the osteogenic cells remains unclear.

The origin of the osteogenic cells and the mechanism driving their differentiation to bone-forming osteoblasts remains to be elucidated. One option would be that the osteogenic cells we consider part of the periosteal cambium layer [32] contribute to bone formation, particularly in upside-down membranes. However, regular placement of the collagen membrane also supports bone formation. At least in theory, in those defects the periosteal cambium layer is shielded away by the dense layer of the collagen membrane; osteogenic cells must consequently originate from the bony walls of the defect site [33] or the dura mater covering the brain. Based on this theory, there should be a considerable difference in outcomes between the two treatment groups, but this is not the case. Hence, we have to propose another model that is based on the migration of type H endothelial cells that carry osteogenic cells and can presumably originate from elevated skin tissue and surrounding bone defects [34]. We can speculate that these endothelial cells can penetrate and spread within the collagen layers of the membrane and provide an equal distribution of the osteogenic cells. These cells then require an osteogenic signal that triggers their differentiation into mature bone-forming osteoblasts, ideally on the surface of the already mineralized collagen fibers that thereby acquire osteoconductive properties. Thus, the studies inspire us to ask where the osteogenic cells come from, how they enter the scaffold structure of the collagen membranes, what drives their osteogenic differentiation, and how the collagen membrane is mineralized in the absence of osteogenic cells.

There are some limitations to our study. First, the clinical relevance of the findings remains a matter of speculation as rat calvarial defects do not fully mimic a clinical scenario of guided bone/tissue regeneration. The findings are thus to be interpreted with caution. It is also unclear whether the rather similar osteoconductive properties of the collagen membrane, independent of the layer alignment, have an impact on the regeneration of bone or other periodontal tissues at all. Other limitations are related to the experimental model. One time point is not ideal to study the early phases of graft consolidation. Indeed, there was a full bone coverage at 4 weeks; no early event was visible such as the possible formation of bone islands that grow and fuse to finally cover the whole defect area. We further did not fix the collagen membrane to the bone. Therefore, micromovements of the membrane with a possible effect on bone regeneration cannot be ruled out. As already mentioned, future studies should reveal the underlying cellular and molecular mechanism to explain the findings of our descriptive research. Future research should also investigate the osteoconductive properties of other collagen membranes of xenogeneic origin. By doing so, we could learn how membrane processing (e.g., defatting, acid/alkaline treatment, dehydration or even disintegration of the collagen fibers, cross-linking) and the properties of the original tissue (e.g., peritoneum, skin, or myocardium) affects the osteoconductive properties of the final products that are applied clinically.

## Conclusion

Within the limitations of this study, our findings support current evidence on the osteoconductive properties of collagen membranes and suggest that bone regeneration is facilitated regardless of membrane layer alignment.

## Supplementary Information

**Additional file 1.** Supplementary Table for Figs. 3b–d and 4b–g

## Data Availability

Source data is provided in Supplementary Table 1.

## References

[1] Hämmerle CH, Jung RE (2003). Bone augmentation by means of barrier membranes. Periodontol.

[2] Jung RE (2018). Alveolar ridge preservation in the esthetic zone. Periodontol.

[3] Sculean A, Nikolidakis D, Schwarz F (2008). Regeneration of periodontal tissues: combinations of barrier membranes and grafting materials - biological foundation and preclinical evidence: a systematic review. J Clin Periodontol.

[4] Benic GI, Hämmerle CH (2014). Horizontal bone augmentation by means of guided bone regeneration. Periodontol.

[5] Bunyaratavej P, Wang HL (2001). Collagen membranes: a review. J Periodontol.

[6] Dimitriou R, Mataliotakis GI, Calori GM, Giannoudis PV (2012). The role of barrier membranes for guided bone regeneration and restoration of large bone defects: current experimental and clinical evidence. BMC Med.

[7] Caballé-Serrano J, et al. Tissue response to a porous collagen matrix used for soft tissue augmentation. Materials (Basel). 2019;12(22):3721. 10.3390/ma12223721 PMC6888327 31718004

[8] Ghanaati S, Schlee M, Webber MJ, Willershausen I, Barbeck M, Balic E, et al. Evaluation of the tissue reaction to a new bilayered collagen matrix in vivo and its translation to the clinic. Biomed Mater. 2011;6(1):015010. 10.1088/1748-6041/6/1/015010. 10.1088/1748-6041/6/1/015010 21239849

[9] You P (2020). Acellular pericardium: a naturally hierarchical, osteoconductive, and osteoinductive biomaterial for guided bone regeneration. J Biomed Mater Res A.

[10] Dahlin C, Linde A, Gottlow J, Nyman S (1988). Healing of bone defects by guided tissue regeneration. Plast Reconstr Surg.

[11] Retzepi M, Donos N (2010). Guided bone regeneration: biological principle and therapeutic applications. Clin Oral Implants Res.

[12] Omar O, Elgali I, Dahlin C, Thomsen P (2019). Barrier membranes: more than the barrier effect?. J Clin Periodontol.

[13] Elgali I, Omar O, Dahlin C, Thomsen P (2017). Guided bone regeneration: materials and biological mechanisms revisited. Eur J Oral Sci.

[14] Turri A, Elgali I, Vazirisani F, Johansson A, Emanuelsson L, Dahlin C, et al. Guided bone regeneration is promoted by the molecular events in the membrane compartment. Biomaterials. 2016;84:167–83. 10.1016/j.biomaterials.2016.01.034. 10.1016/j.biomaterials.2016.01.034 26828682

[15] Kuchler U, Rybaczek T, Dobask T, Heimel P, Tangl S, Klehm J, et al. Bone-conditioned medium modulates the osteoconductive properties of collagen membranes in a rat calvaria defect model. Clin Oral Implants Res. 2018;29(4):381–8. 10.1111/clr.13133. 10.1111/clr.13133 29453780

[16] Strauss FJ, et al. Acid bone lysates reduce bone regeneration in rat calvaria defects. J Biomed Mater Res A. 2021;109(5):659-65. 10.1002/jbm.a.37050. 10.1002/jbm.a.37050 PMC7984281 32608132

[17] Nudelman F, Lausch AJ, Sommerdijk NAJM, Sone ED (2013). In vitro models of collagen biomineralization. J Struct Biol.

[18] Wang J, Glimcher MJ (1999). Characterization of matrix-induced osteogenesis in rat calvarial bone defects: I. Differences in the cellular response to demineralized bone matrix implanted in calvarial defects and in subcutaneous sites. Calcif Tissue Int.

[19] Gruber R, Mayer C, Bobacz K, Krauth MT, Graninger W, Luyten FP, et al. Effects of cartilage-derived morphogenetic proteins and osteogenic protein-1 on osteochondrogenic differentiation of periosteum-derived cells. Endocrinology. 2001;142(5):2087–94. 10.1210/endo.142.5.8163. 10.1210/endo.142.5.8163 11316776

[20] Wang J, Gao Y, Cheng P, Li D, Jiang H, Ji C, et al. CD31hiEmcnhi vessels support new trabecular bone formation at the frontier growth area in the bone defect repair process. Sci Rep. 2017;7(1):4990. 10.1038/s41598-017-04150-5. 10.1038/s41598-017-04150-5 PMC5504063 28694480

[21] Petrie Aronin CE, Cooper JA, Sefcik LS, Tholpady SS, Ogle RC, Botchwey EA (2008). Osteogenic differentiation of dura mater stem cells cultured in vitro on three-dimensional porous scaffolds of poly(epsilon-caprolactone) fabricated via co-extrusion and gas foaming. Acta Biomater.

[22] Fahmy RA, Kotry GS, Ramadan OR (2018). Periodontal regeneration of dehisence defects using a modified perforated collagen membrane. A comparative experimental study. Future Dent J.

[23] Kim TH, Oh SH, Na SY, Chun SY, Lee JH (2012). Effect of biological/physical stimulation on guided bone regeneration through asymmetrically porous membrane. J Biomed Mater Res A.

[24] Górski B, Jalowski S, Górska R, Zaremba M (2018). Treatment of intrabony defects with modified perforated membranes in aggressive periodontitis: a 12-month randomized controlled trial. Clin Oral Investig.

[25] Gamal AY, Iacono VJ (2013). Enhancing guided tissue regeneration of periodontal defects by using a novel perforated barrier membrane. J Periodontol.

[26] Schindelin J, Arganda-Carreras I, Frise E, Kaynig V, Longair M, Pietzsch T, et al. Fiji: an open-source platform for biological-image analysis. Nat Methods. 2012;9(7):676–82. 10.1038/nmeth.2019. 10.1038/nmeth.2019 PMC3855844 22743772

[27] Issa DR, Abdel-Ghaffar KA, al-Shahat MA, Hassan AAA, Iacono VJ, Gamal AY (2020). Guided tissue regeneration of intrabony defects with perforated barrier membranes, simvastatin, and EDTA root surface modification: a clinical and biochemical study. J Periodontal Res.

[28] Gamal AY, al-Berry NN, Hassan AA, Rashed LA, Iacono VJ (2017). In vitro evaluation of the human gingival fibroblast/gingival mesenchymal stem cell dynamics through perforated guided tissue membranes: cell migration, proliferation and membrane stiffness assay. J Periodontal Res.

[29] Górski B, Jalowski S, Górska R, Zaremba M (2019). Treatment of intrabony defects with modified perforated membranes in aggressive periodontitis: subtraction radiography outcomes, prognostic variables, and patient morbidity. Clin Oral Investig.

[30] Gamal AY, Aziz M, Salama MH, Iacono VJ (2014). Gingival crevicular fluid bone morphogenetic protein-2 release profile following the use of modified perforated membrane barriers in localized intrabony defects: a randomized clinical trial. J Int Acad Periodontol.

[31] Khorsand B, Elangovan S, Hong L, Kormann MSD, Salem AK (2019). A bioactive collagen membrane that enhances bone regeneration. J Biomed Mater Res B Appl Biomater.

[32] Dwek JR (2010). The periosteum: what is it, where is it, and what mimics it in its absence?. Skelet Radiol.

[33] Hämmerle CH (1995). Temporal dynamics of healing in rabbit cranial defects using guided bone regeneration. J Oral Maxillofac Surg.

[34] Sivaraj KK, Adams RH (2016). Blood vessel formation and function in bone. Development.

